# Crystalloid-Dependent Modulation of Aquaporins and Molecular Stress Pathways During Acute Normovolemic Hemodilution in Rat Heart and Aorta

**DOI:** 10.3390/ijms27219498

**Published:** 2026-10-24

**Authors:** Kerem Erkalp, Aslıhan Şengelen, Serdar Demirgan, Yunus Aksüt, Evren Önay-Uçar, Nermin Yelmen

**Affiliations:** 1 Department of Anesthesiology and Reanimation, Institute of Cardiology, Istanbul University-Cerrahpaşa, Istanbul 34098, Türkiye; 2 Department of Physiology, Cerrahpaşa Faculty of Medicine, Istanbul University-Cerrahpaşa, Istanbul 34098, Türkiye; nermink@iuc.edu.tr or nerminyelmen@aydin.edu.tr; 3 Department of Molecular Biology and Genetics, Faculty of Engineering and Natural Sciences, Biruni University, Istanbul 34010, Türkiye; 4 Department of Molecular Biology and Genetics, Faculty of Science, Istanbul University, Istanbul 34134, Türkiye; aksut.yns@gmail.com (Y.A.); evrenonay@istanbul.edu.tr (E.Ö.-U.); 5 Clinic of Anesthesiology and Reanimation, University of Health Sciences, Bağcılar Training and Research Hospital, Istanbul 34200, Türkiye; serdardemirgan@hotmail.com; 6 Center for Research and Practice in Biotechnology and Genetic Engineering, Istanbul University, Istanbul 34134, Türkiye; 7 Department of Physiology, Faculty of Medicine, Istanbul Aydın University, Istanbul 34295, Türkiye

**Keywords:** hemodilution, balanced vs. unbalanced crystalloids, aquaporins (AQPs), cardiovascular stress responses, rat model

## Abstract

Acute normovolemic hemodilution (ANH) maintains circulating volume with replacement fluids, but its harmful effects and whether crystalloid composition alters cardiovascular tissue stress remain unclear. This study compared normal saline (NS) and Ringer’s lactate (RL) during ANH to determine fluid-dependent molecular effects in the rat heart and aorta. In this study, twenty-four male rats were assigned to control (no procedure), sham (anesthesia only), normal saline (NS)–ANH, or Ringer lactate (RL)–ANH groups (*n* = 6/group). ANH was performed using a fixed 3:1 crystalloid-to-blood replacement ratio. Heart and aorta tissues were collected 24 h later. Apoptotic, inflammatory, oxidative, heat-shock, and aquaporin proteins were quantified by immunoblotting. Hematocrit decreased similarly in both ANH groups. Compared with NS-ANH, RL-ANH reduced apoptosis in both tissues and reduced cardiac TNF-α levels and aortic NF-κB p65 and TNF-α, but not cardiac NF-κB p65. Cardiac 4-HNE and MDA increased similarly with both fluids, whereas aortic lipid peroxidation markers were lower with RL. Hsp70 responses were comparable, but Hsp90 was consistently higher with RL. RL-ANH also induced distinct aquaporin remodeling, with higher cardiac AQP1 and aortic AQP1, AQP3, and AQP4 levels than NS-ANH, although aortic AQP3 remained significantly lower than in the sham group. Correlation analysis showed tissue-specific aquaporin–injury coupling: cardiac AQP1 was associated with reduced apoptosis and inflammation, cardiac AQP3 with increased oxidative injury, and aortic AQPs with predominantly inverse injury relationships. Consequently, crystalloid composition influenced cardiovascular adaptation to ANH. RL reduced apoptotic and vascular inflammatory activity, limited aortic oxidative stress, and produced distinct Hsp90 and aquaporin profiles. Tissue-specific aquaporin–stress associations further underscored mechanistic divergence between fluids. These molecular signatures reveal early shifts in stress-response pathways and highlight the biological importance of selecting the appropriate crystalloid during ANH.

## 1. Introduction

Acute normovolemic hemodilution (ANH) is a widely used blood conservation strategy that reduces exposure to allogeneic transfusion during procedures associated with substantial blood loss [1,2]. By withdrawing autologous whole blood and replacing the removed volume with crystalloid or colloid solutions, ANH maintains intravascular volume while producing a controlled reduction in hematocrit and blood viscosity [3,4]. Although ANH is generally considered safe and effective, accumulating evidence suggests that its physiological effects extend beyond simple volume replacement and may be influenced by the characteristics of the replacement fluid [5,6].

The biological consequences of ANH depend not only on the degree of hemodilution but also on the physicochemical properties of the administered solution. Isotonic saline, one of the most commonly used crystalloids, contains supraphysiologic chloride concentrations and lacks buffering capacity, potentially contributing to acid–base disturbances and endothelial dysfunction. In contrast, Ringer’s lactate contains lower chloride levels and a metabolizable buffer, making it a more physiologically balanced solution [7]. Although clinical studies comparing balanced crystalloids with saline have reported conflicting outcomes, the SMART trial demonstrated modest benefits associated with balanced solutions, whereas the PLUS trial found no significant differences in mortality or kidney injury [8,9]. These findings suggest that fluid composition may influence tissue responses independently of volume expansion and that such effects may be context-dependent.

Cardiovascular tissues may be especially sensitive to hemodilution-induced alterations. Changes in oxygen delivery, blood viscosity, electrolyte balance, and osmotic gradients may activate oxidative, inflammatory, and apoptosis-related molecular pathways that could contribute to tissue injury [10,11,12]. Experimental evidence further suggests that balanced crystalloids may attenuate some of these adverse effects compared to saline [13], although data specifically related to ANH remain limited.

Among the molecular systems potentially involved in ANH-induced stress responses, aquaporins (AQPs) are of particular interest. AQP1, AQP3, and AQP4 are expressed in cardiac and vascular tissues and contribute to water transport, tissue hydration, edema formation, and cellular adaptation to osmotic stress [14,15]. Prior studies have shown that fluid resuscitation and hemodynamic disturbances can modify AQP expression in brain, pulmonary, and endothelial tissues [5,16,17], suggesting that AQPs may be sensitive to shifts in ionic gradients and plasma osmolality. In parallel, ANH may activate broader cellular stress pathways; rapid dilution of blood components and electrolyte imbalance may disrupt endothelial homeostasis and promote inflammatory and apoptosis-associated signaling, while metabolic strain may enhance stress responses and oxidative by-product formation [6,10,11,12,18]. However, these molecular responses do not establish inflammatory cell infiltration or histological cell death without morphological confirmation [19,20]. Together, these observations highlight AQPs as potential mediators of the coordinated inflammatory, apoptotic, and oxidative stress profile elicited by ANH, yet whether different crystalloid solutions differentially influence cardiovascular AQP regulation remains unknown.

In our previous experimental study, ANH induced organ-specific alterations in aquaporin expression and activated inflammatory and apoptotic pathways [21]. These findings suggest that hemodilution triggers distinct molecular stress responses, but the contribution of replacement-fluid composition remains unclear. Ringer’s lactate and isotonic saline differ substantially in electrolyte profiles, chloride loads, and buffering capacities, factors that may influence endothelial stability, oxidative stress, and water-channel regulation [22,23]. Therefore, the present study aimed to compare the effects of Ringer’s lactate and isotonic saline on early molecular responses in heart and aortic tissues during ANH. We hypothesized that Ringer’s lactate would be associated with a less pronounced inflammatory, oxidative, and apoptotic response than isotonic saline. By evaluating markers of apoptosis, inflammation, oxidative injury, cellular stress adaptation, and aquaporin regulation, this study seeks to clarify fluid-specific biological effects and provide mechanistic insights to optimize perioperative hemodilution strategies.

## 2. Results

### 2.1. Hemodilution Model Validation and Bioparameters

Body weight and estimated blood volume were comparable across all groups (*p* = 0.9993 and *p* = 0.2417, respectively; Table 1). Median estimated blood volumes ranged from 27.85–29.57 mL, with no significant differences among the control, sham, NS-ANH, and RL-ANH groups. The volume of blood collected during hemodilution was similar between NS-ANH and RL-ANH (11.80 vs. 11.43 mL, *p* = 0.7345).

Baseline hematocrit (HCT) values did not differ among groups (*p* = 0.8442). HCT remained stable in the control and sham groups over 24 h (*p* = 0.5543 and *p* = 0.3030); both ANH strategies produced a marked reduction: NS-ANH decreased HCT from 39.00% to 24.50%, and RL-ANH from 40.00% to 26.50% (both *p* < 0.0001). Consequently, a significant intergroup difference emerged at 24 h (*p* < 0.0001), confirming successful induction of acute normovolemic hemodilution.

### 2.2. Apoptotic and Inflammatory Activation Under Crystalloid-Dependent ANH

**Figure 1:**
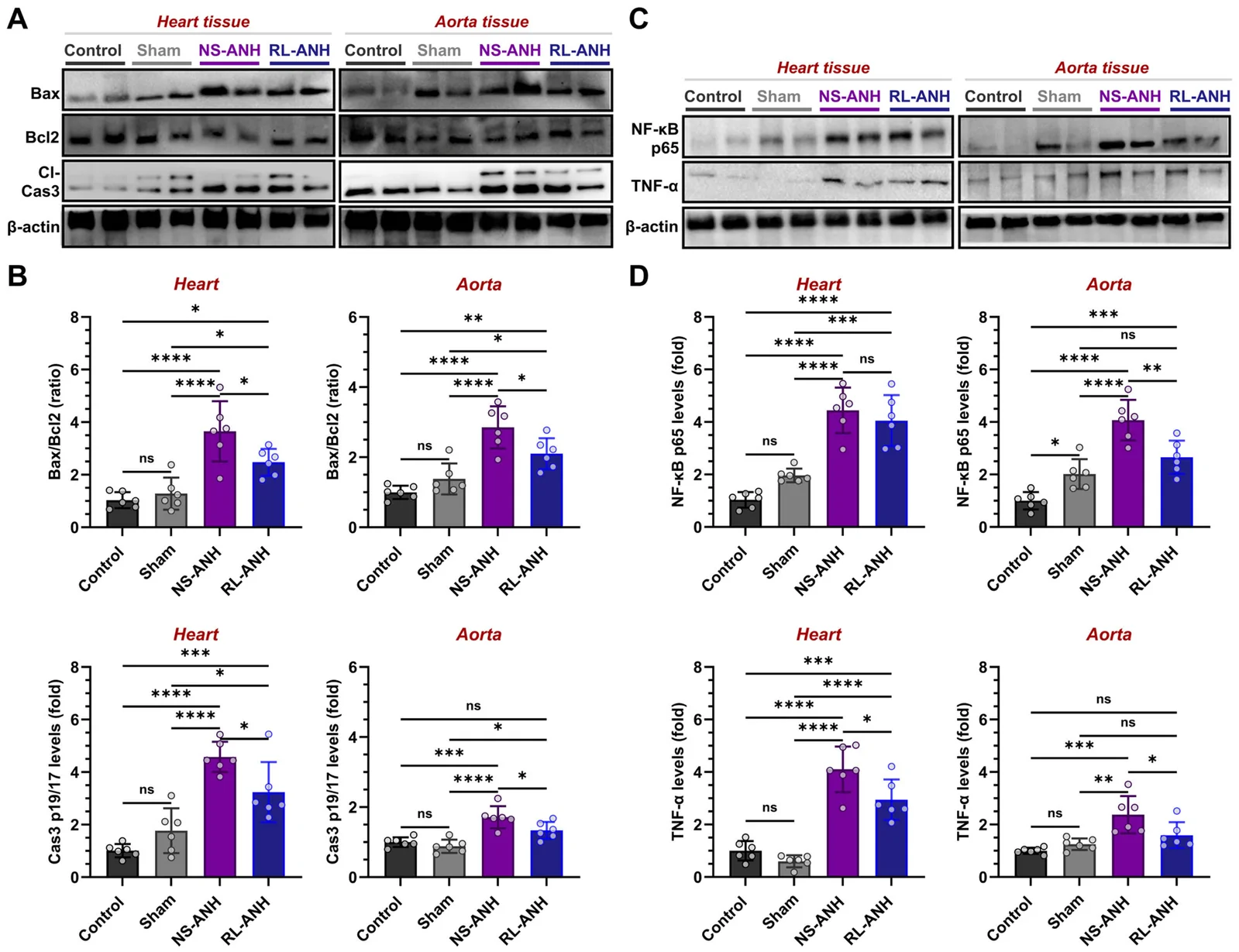
Modulation of apoptotic and inflammatory signaling in heart and aorta following crystalloid-dependent ANH. Assessments were conducted on the control, sham, normal-saline (NS)-ANH, or Ringer’s-lactate (RL)-ANH groups. (**A**) Western blot analysis of Bax, Bcl2, and cleaved caspase-3 and (**B**) their quantification. (**C**) Western blot analysis of NF-κB p65 and TNF-α and (**D**) their quantification. β-actin served as loading control. Data are mean ± SD (*n* = 6). * *p* < 0.05, ** *p* < 0.01, *** *p* < 0.001, **** *p* < 0.0001; ns, not significant.

Apoptotic signaling was assessed using the Bax/Bcl2 ratio and cleaved caspase-3 p19/17 (Figure 1A,B). Control and sham groups showed comparable levels (*p* > 0.05). In cardiac tissue, NS-ANH markedly increased the Bax/Bcl2 ratio (2.85-fold) and cleaved caspase-3 (2.59-fold) relative to sham (both *p* < 0.0001). RL-ANH also elevated these markers (1.93- and 1.83-fold; *p* = 0.0410 and *p* = 0.0192), but to a lesser extent. Direct comparison revealed significantly lower apoptosis-associated marker levels under RL-ANH (1.47- and 1.42-fold reductions vs. NS-ANH; *p* = 0.0453 and *p* = 0.0344). Aortic tissue exhibited a similar pattern, with NS-ANH producing higher apoptosis-associated marker levels than RL-ANH (1.36-fold, *p* = 0.0390 for Bax/Bcl2; 1.28-fold, *p* = 0.0460 for active caspase-3).

Inflammatory signaling was evaluated by measuring NF-κB p65 and TNF-α levels (Figure 1C,D). Cardiac NF-κB p65 and TNF-α increased substantially under NS-ANH (2.26- and 6.84-fold; both *p* < 0.0001) and under RL-ANH (2.06- and 4.91-fold; *p* = 0.0002 and *p* < 0.0001). TNF-α was significantly lower in RL-ANH than in NS-ANH (1.39-fold, *p* = 0.0204). In the aorta, NS-ANH induced robust increases in NF-κB p65 (2.02-fold) and TNF-α (1.90-fold), whereas RL-ANH did not differ from sham (*p* > 0.05). RL-ANH reduced cardiac TNF-α levels, but not NF-κB p65 levels, and reduced both markers in the aorta compared with NS-ANH.

### 2.3. Oxidative and Glycoxidative Damage Profiles Under Crystalloid-Dependent ANH

**Figure 2:**
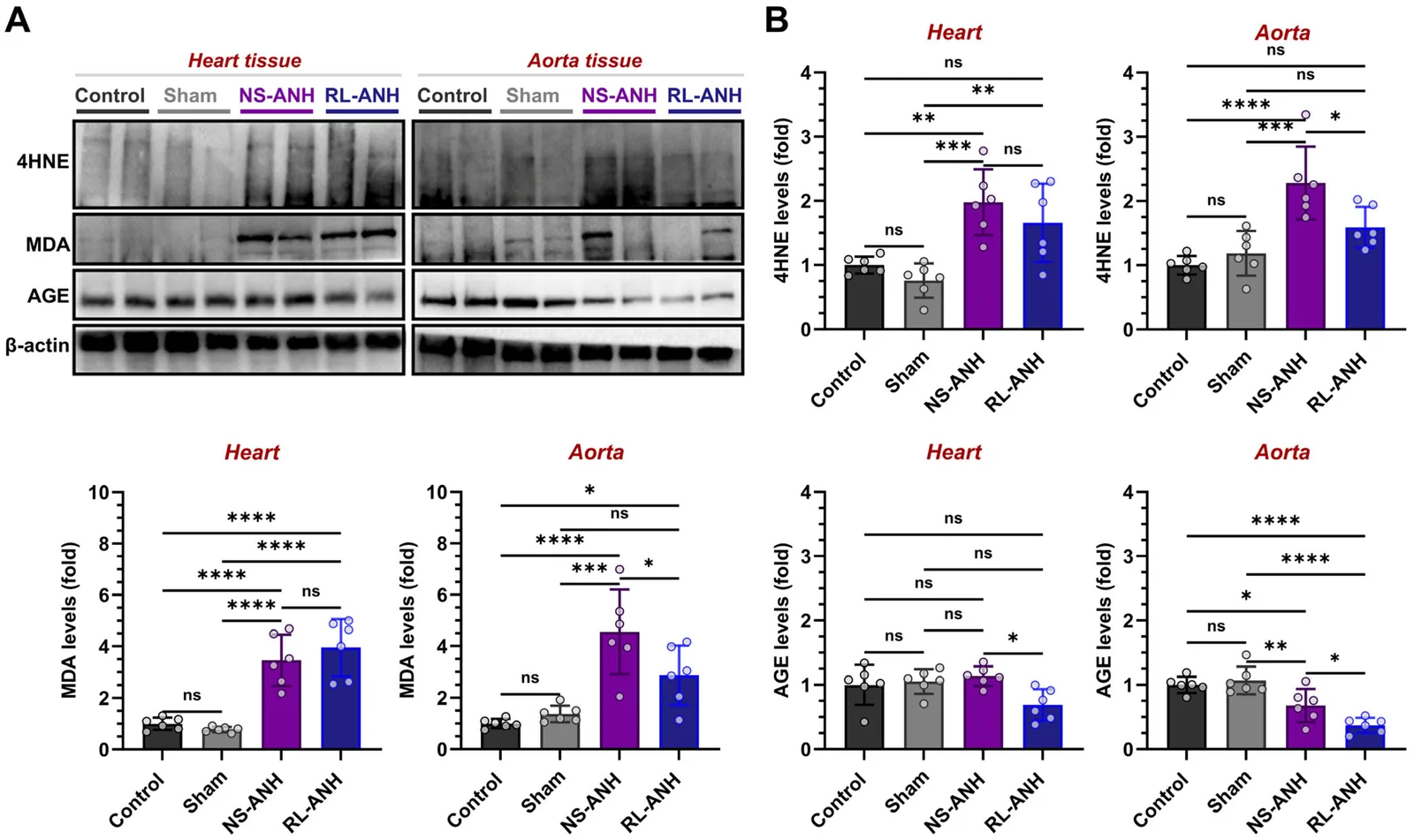
Oxidative and glycoxidative injury profiles in heart and aorta following crystalloid-dependent ANH. Assessments were conducted on the control, sham, normal-saline (NS)-ANH, or Ringer’s-lactate (RL)-ANH groups. (**A**) Western blot analysis of 4-HNE, MDA, and AGE and (**B**) their quantification. β-actin served as loading control. Data are mean ± SD (*n* = 6). * *p* < 0.05, ** *p* < 0.01, *** *p* < 0.001, **** *p* < 0.0001; ns, not significant.

Lipid peroxidation (4-HNE and MDA) and glycation/glycoxidation-related changes (AGE) were assessed (Figure 2A,B). The control and sham groups showed similar levels (*p* > 0.05). In cardiac tissue, NS-ANH markedly increased 4-HNE (2.61-fold, *p* = 0.0004) and MDA (4.47-fold, *p* < 0.0001), whereas AGE remained unchanged. RL-ANH produced comparable increases in 4-HNE (2.18-fold, *p* = 0.0078) and MDA (5.10-fold, *p* < 0.0001). AGE differed between ANH groups (1.64-fold higher in NS-ANH, *p* = 0.0164), whereas 4-HNE and MDA did not. In aortic tissue, NS-ANH increased 4-HNE (1.92-fold, *p* = 0.0003) and MDA (3.32-fold, *p* = 0.0001), while decreasing AGE (1.82-fold, *p* = 0.0091). RL-ANH did not alter 4-HNE or MDA relative to sham but significantly reduced AGE (2.87-fold, *p* < 0.0001). RL-ANH generally showed lower lipid peroxidation and AGE-related markers than NS-ANH, particularly in aortic tissue.

### 2.4. Heat Shock Protein Response to Crystalloid-Driven ANH

**Figure 3:**
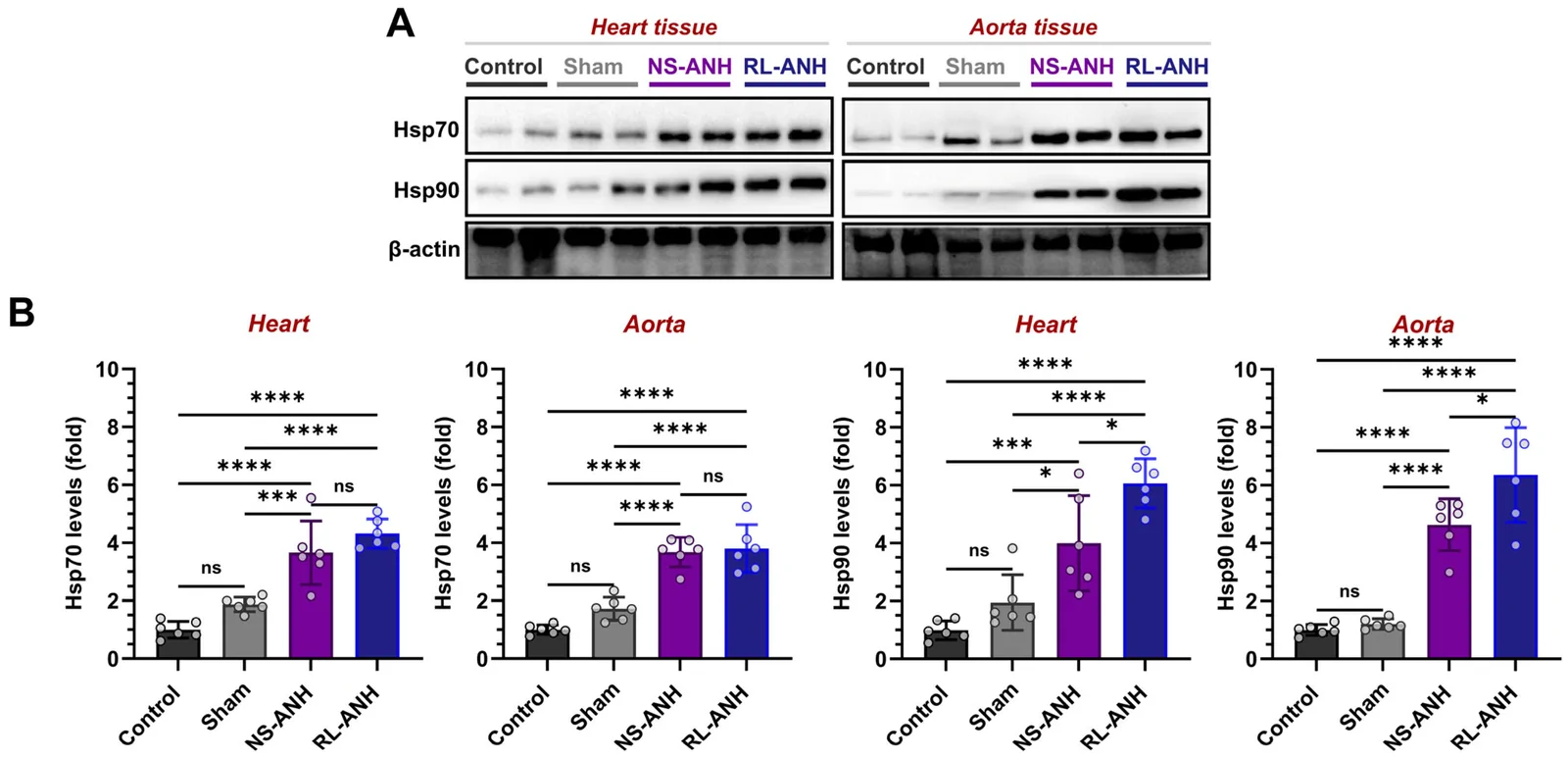
Heat-shock protein responses in heart and aorta following crystalloid-dependent ANH. Assessments were conducted on the control, sham, normal-saline (NS)-ANH, or Ringer’s-lactate (RL)-ANH groups. (**A**) Western blot analysis of Hsp70 and Hsp90 levels and (**B**) their quantification. β-actin served as loading control. Data are mean ± SD (*n* = 6). * *p* < 0.05, *** *p* < 0.001, **** *p* < 0.0001; ns, not significant.

The cellular stress response was assessed by measuring Hsp70 and Hsp90 levels (Figure 3A,B). Control and sham groups did not differ in either tissue (*p* > 0.05). In cardiac tissue, NS-ANH increased Hsp70 (1.96-fold, *p* = 0.0005) and Hsp90 (2.05-fold, *p* = 0.0153). RL-ANH induced even greater increases (2.31-fold and 3.11-fold, respectively; both *p* < 0.0001). Hsp90 was significantly higher in RL-ANH than in NS-ANH (1.52-fold, *p* = 0.0140). In aortic tissue, NS-ANH elevated Hsp70 (2.14-fold) and Hsp90 (3.85-fold), whereas RL-ANH produced greater increases (2.21-fold and 5.28-fold; both *p* < 0.0001). No difference in Hsp70 was detected between the two ANH groups (*p* > 0.05), whereas Hsp90 was 1.37-fold higher in RL-ANH than in NS-ANH (*p* = 0.0224).

### 2.5. Aquaporin Regulation Under Crystalloid-Dependent ANH

**Figure 4:**
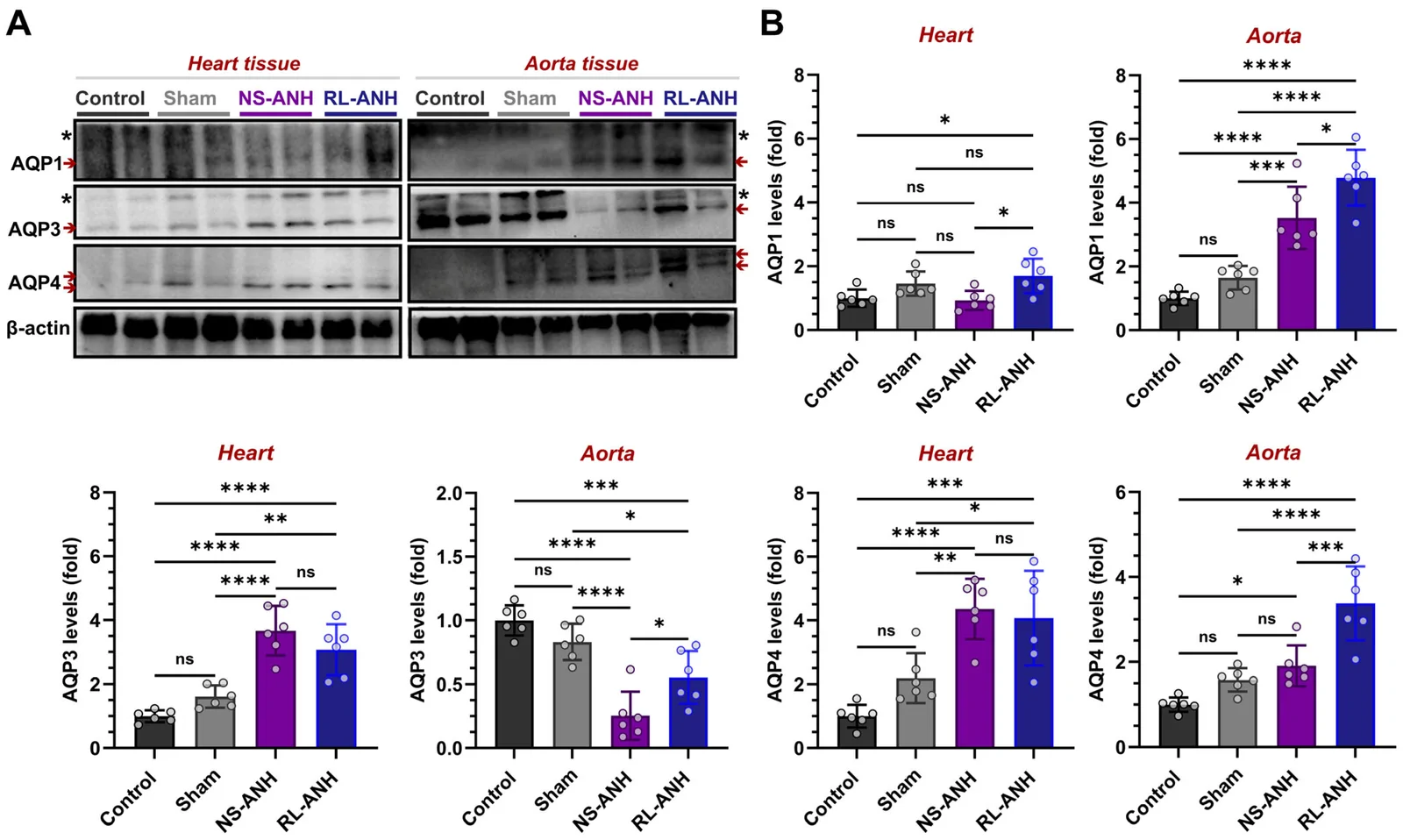
Modulation of aquaporin expression in heart and aorta following crystalloid-dependent ANH. Assessments were conducted on the control, sham, normal-saline (NS)-ANH, or Ringer’s-lactate (RL)-ANH groups. Higher-molecular-weight AQP1 and AQP3 bands, indicating putative glycosylated isoforms, are marked with asterisks (*), and red arrows indicate non-glycosylated protein bands. (**A**) Western blot analysis of AQP1, AQP3, and AQP4 and (**B**) their quantification. β-actin served as loading control. Data are mean ± SD (*n* = 6). * *p* < 0.05, ** *p* < 0.01, *** *p* < 0.001, **** *p* < 0.0001; ns, not significant.

AQP1, AQP3, and AQP4 expression was examined to characterize fluid-dependent modulation of water transport pathways (Figure 4A,B). Control and sham groups showed comparable levels (*p* > 0.05). In cardiac tissue, NS-ANH markedly upregulated AQP3 (2.27-fold, *p* < 0.0001) and AQP4 (1.99-fold, *p* = 0.0053), whereas AQP1 remained unchanged. RL-ANH markedly increased AQP1 (2.59-fold, *p* < 0.0001), AQP3 (1.90-fold, *p* = 0.0020), and AQP4 (1.86-fold, *p* = 0.0164) relative to sham. AQP1 was significantly higher in RL-ANH than in NS-ANH (1.82-fold, *p* = 0.0126). In aortic tissue, NS-ANH increased AQP1 levels (2.14-fold, *p* = 0.0007) and markedly reduced AQP3 levels (3.27-fold, *p* < 0.0001), with no change in AQP4 levels compared to sham. RL-ANH elevated AQP1 (2.91-fold) and AQP4 levels (2.14-fold; both *p* < 0.0001) while reducing AQP3 (1.50-fold, *p* = 0.0446) relative to sham. RL-ANH produced higher expression of all three AQPs compared with NS-ANH (AQP1: 1.36-fold, *p* = 0.0233; AQP3: 2.18-fold, *p* = 0.0271; AQP4: 1.77-fold, *p* = 0.0005).

Remarkably, in both heart and aortic tissues, Western blots revealed additional higher-molecular-weight AQP1 and AQP3 bands, marked with asterisks in the figures. These bands likely represent glycosylated aquaporin isoforms that migrate at higher molecular weights due to post-translational modification. Although densitometric analyses focused on the non-glycosylated forms, the glycosylated AQP species were consistently detectable and appeared more prominent under ANH conditions.

### 2.6. Correlation Analysis Between Aquaporins and Apoptotic, Inflammatory, Oxidative, and Stress Response Markers

**Figure 5:**
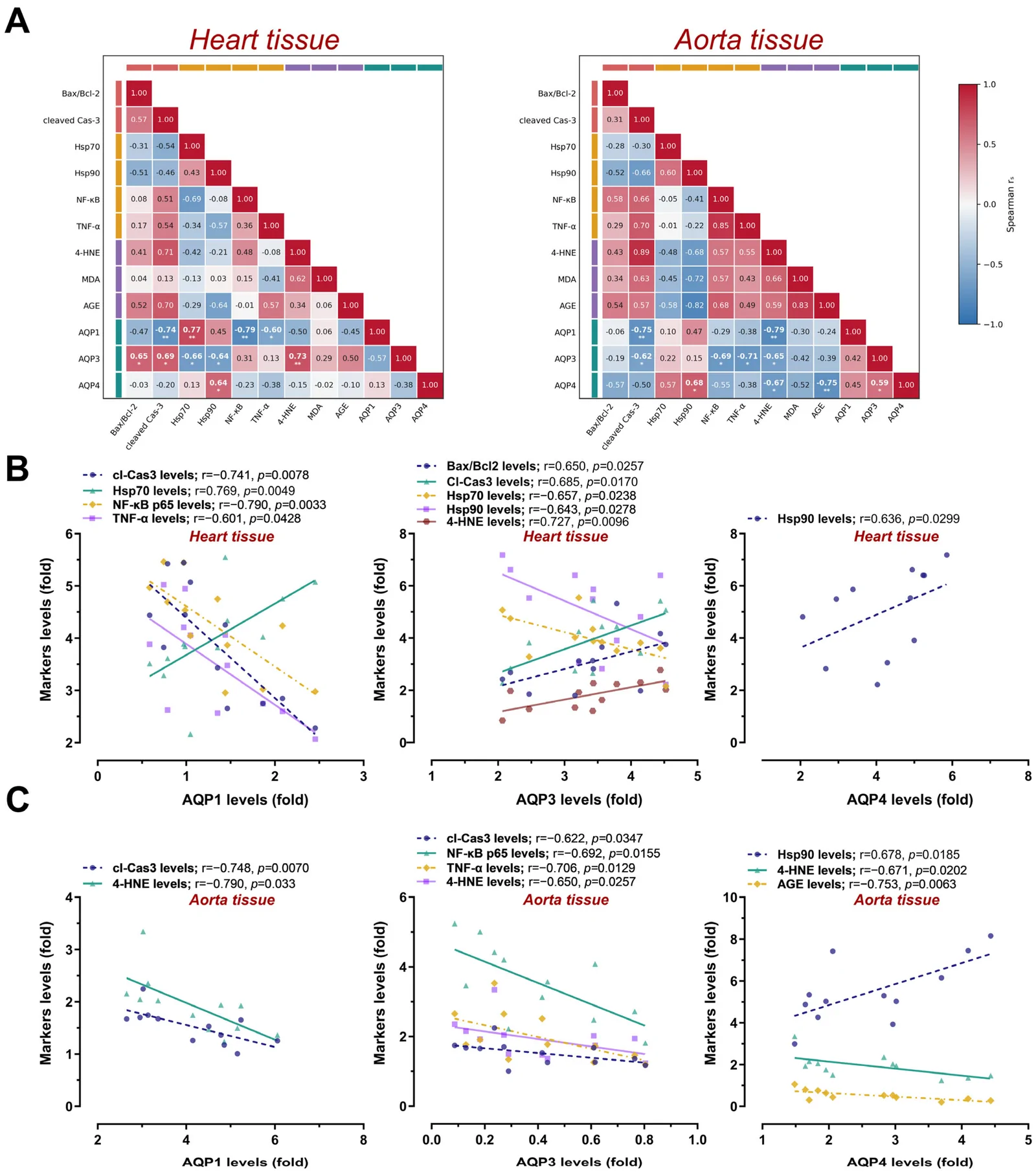
Tissue-specific correlations between aquaporin expression and apoptotic, inflammatory, oxidative, and stress-response markers under crystalloid-dependent ANH. Correlation analysis (*n* = 12) was performed using pooled NS-ANH and RL-ANH results. (**A**) Correlation matrices showing relationships between AQP1, AQP3, AQP4, and apoptotic, inflammatory, oxidative, and heat-shock markers in heart and aorta. (**B**) Significant correlations in heart tissue. (**C**) Significant correlations in aortic tissue. Spearman’s correlation coefficients were interpreted as weak (r = 0.20–0.39), moderate (r = 0.40–0.59), strong (r = 0.60–0.79), and very strong (r = 0.80–1.0). * *p* < 0.05, ** *p* < 0.01.

Spearman’s rank-correlation analysis was conducted in the pooled ANH cohort to examine the relationships between aquaporin expression and the markers of apoptosis, inflammation, oxidative damage, and cellular stress (Figure 5A–C). The correlation architecture differed markedly between cardiac and aortic tissues, indicating tissue-specific aquaporin–injury coupling.

In cardiac tissue, AQP1 expression showed inverse associations with apoptotic (cleaved caspase-3) and inflammatory (NF-κB p65 and TNF-α) markers, and a positive association with Hsp70. Higher AQP1 expression correlated inversely with cleaved caspase-3 (r = −0.741, *p* = 0.0078), NF-κB p65 (r = −0.790, *p* = 0.0033), and TNF-α (r = −0.601, *p* = 0.0428), while positively correlating with Hsp70 (r = 0.769, *p* = 0.0049). In contrast, higher AQP3 expression was positively correlated with the Bax/Bcl2 ratio (r = 0.650, *p* = 0.0257), cleaved caspase-3 (r = 0.685, *p* = 0.0170), and 4-HNE (r = 0.727, *p* = 0.0096). AQP3 was inversely correlated with Hsp70 (r = −0.657, *p* = 0.0238) and Hsp90 (r = −0.643, *p* = 0.0278). Cardiac AQP4 showed a single significant association, positively correlating with Hsp90 (r = 0.636, *p* = 0.0299).

In aortic tissue, aquaporin–injury relationships were more uniformly inverse. AQP1 was negatively correlated with cleaved caspase-3 (r = −0.748, *p* = 0.0070) and 4-HNE (r = −0.790, *p* = 0.0033). AQP3 also showed inverse correlations with cleaved caspase-3 (r = −0.622, *p* = 0.0347), NF-κB p65 (r = −0.692, *p* = 0.0155), TNF-α (r = −0.706, *p* = 0.0129), and 4-HNE (r = −0.650, *p* = 0.0257). AQP4 showed a mixed pattern: it correlated positively with Hsp90 (r = 0.678, *p* = 0.0185) but inversely with 4-HNE (r = −0.671, *p* = 0.0202) and AGE (r = −0.753, *p* = 0.0063).

Overall, cardiac AQP1 was associated with reduced apoptotic and inflammatory signaling, whereas cardiac AQP3 was positively associated with apoptosis and lipid peroxidation. In the aorta, AQP1, AQP3, and AQP4 were predominantly inversely associated with markers of apoptosis, inflammation, or oxidative damage, while AQP4 expression paralleled the Hsp90 response.

## 3. Discussion

Acute normovolemic hemodilution (ANH) preserves intravascular volume while lowering hematocrit, thereby altering oxygen delivery, cardiac workload, and vascular shear stress [24,25]. Mechanical forces, particularly blood flow-induced shear stress, are important regulators of endothelial responses [26]. Our previous report demonstrated tissue-specific apoptotic, inflammatory, and aquaporin responses to ANH [21], but it remained unclear whether these responses depended on the replacement fluid. This question is clinically relevant because normal saline (NS) and Ringer’s lactate (RL) differ in chloride concentration, buffering capacity, and ionic composition, and their physiological effects vary across experimental and clinical settings [27,28]. The present study addressed this gap by comparing NS- and RL-based ANH and characterizing 24 h molecular responses in the rat heart and aorta.

The ANH model was successfully applied to both fluid groups, with comparable baseline hematocrit and blood-withdrawal volumes. The fixed 3:1 crystalloid-to-blood replacement produced hemodilution at 24 h, consistent with our previous report [21] and with another experimental ANH protocol [29]. Although clinical ANH typically uses lower crystalloid volumes and relies on reinfusion to recover hematocrit [30,31], the present model provides a controlled platform for evaluating fluid-dependent molecular responses.

Apoptotic and inflammatory responses showed clear fluid-dependent differences. NS-ANH consistently produced higher Bax/Bcl2 ratios and cleaved caspase-3 levels than RL-ANH in both tissues, indicating a more pronounced apoptosis-associated molecular response. RL-ANH attenuated, but did not eliminate, apoptotic signaling, consistent with evidence that RL resuscitation reduces myocardial Bax and caspase activation after trauma-hemorrhagic shock [13] and with findings in heatstroke rats, where NS induced greater apoptosis, TNF-α production, and structural injury than RL [27]. These observations are further supported by vascular studies showing that NS produces greater endothelial permeability and leukocyte adhesion than RL after experimental hemorrhage [32] and that direct exposure of vascular tissue to acidic, unbuffered saline activates endothelial injury and NF-κB-dependent inflammatory signaling, whereas buffered solutions such as Plasma-Lyte do not elicit this response [24]. Together, these data suggest that stronger aortic apoptotic and inflammatory activation under NS-ANH may reflect direct vascular sensitivity to crystalloid composition and extend our previous finding that balanced-crystalloid ANH triggered apoptotic and inflammatory responses in both rat heart and aorta [21]. Nevertheless, interpretation of NF-κB p65 requires caution because total protein levels do not reflect phosphorylation or nuclear translocation, and NF-κB and TNF-α exhibit different temporal dynamics [33]. Thus, NS-ANH showed more pronounced apoptosis-associated responses in both tissues and higher aortic inflammatory marker levels, although further histological studies are needed to confirm their tissue-level significance.

Oxidative and glycoxidative responses showed tissue-specific fluid effects. In the heart, both NS-ANH and RL-ANH increased 4-HNE and MDA, indicating that RL did not prevent the ANH-associated increase in cardiac lipid peroxidation markers. This interpretation aligns with a report of increased systemic and tissue MDA, hypoxia, and inflammatory injury during normovolemic hemodilution [34]. In contrast, NS-ANH increased aortic 4-HNE and MDA, whereas RL-ANH remained comparable to sham and showed lower levels than NS-ANH. This supports a tissue-specific oxidative response and aligns with evidence that NS causes greater endothelial and microvascular dysfunction than RL after hemorrhage [32,35]. AGE responses did not parallel lipid peroxidation. Cardiac AGE remained unchanged, whereas aortic AGE decreased in both fluids. Because vascular AGE formation occurs over days rather than hours [36], 24 h AGE levels likely reflect acute matrix-related changes rather than oxidative injury. Thus, AGE findings should be interpreted separately from lipid peroxidation markers, as they may reflect distinct glycation/glycoxidation-related processes.

Both fluids increased Hsp70 and Hsp90 levels in the heart and aorta, indicating a shared proteostatic stress response, consistent with the established roles of heat shock proteins in proteostasis and cellular stress adaptation [37]. Hsp70 did not differ between fluids, whereas Hsp90 was consistently higher with RL. Because Hsp70 and Hsp90 perform linked but non-interchangeable functions within the chaperone cycle [38], their divergent profiles may reflect differential activation thresholds. The higher Hsp90 response with RL coincided with lower apoptotic and aortic lipid-peroxidation signals, suggesting enhanced stress adaptation. However, HSP abundance can represent either protection or injury depending on context. Hsp70 levels may be associated with myocardial protection, whereas Hsp90 levels may be related to operative stress and to troponin release [18]. Moreover, ROS generation and apoptosis can coexist with increased Hsp70/Hsp90, and experimental enhancement of the heat-shock response reduces both outcomes via PI3K/Akt signaling [39]. Because measurements of HSF1 activation, Hsp90 client interactions, or pathway inhibition were not performed, the higher Hsp90 abundance after RL suggests an associated adaptive response rather than a protective mechanism.

Aquaporin expression showed fluid-dependent changes, which may reflect differences in water transport regulation. Both NS-ANH and RL-ANH increased cardiac AQP3 and aortic AQP1, with reciprocal suppression of aortic AQP3, consistent with our previous balanced-crystalloid ANH findings [21]. However, RL-ANH produced higher cardiac AQP1 and a more pronounced aortic AQP4 response, whereas NS-ANH more closely resembled the prior balanced-crystalloid profile. These findings suggest that AQP3 changes may be directly associated with the hemodilution process, whereas AQP1 and AQP4 are more sensitive to fluid composition. Mechanistically, fluid-dependent AQP regulation aligns with known biological principles. Distinct pulmonary AQP1/AQP5 and cerebral AQP4 responses have been reported after hemorrhagic shock, depending on the resuscitation fluid [5,40]. AQP1 also mediates hydrostatic-pressure-driven water transport across the aortic endothelium, and osmotic stimulation induces calcium/calmodulin-dependent AQP1 membrane recruitment [41,42]. Because NS and RL differ in chloride concentration, osmolarity, buffering, and ionic composition, their AQP profiles likely reflect combined ionic, osmotic, acid–base, and hemodynamic influences rather than a single mechanism. In addition, immunoblotting analysis revealed additional higher-molecular-weight AQP1 and AQP3 bands in both tissues, consistent with glycosylated aquaporin isoforms. Glycosylation is a known post-translational modification that affects AQP folding, membrane trafficking, and channel stability [43,44]. Although densitometric analyses focused on non-glycosylated forms, the reproducible detection of glycosylated AQP species, particularly under ANH conditions, suggests that replacement-fluid composition may modulate aquaporin biology not only by altering the protein abundance but also by changing the post-translational processing. This interpretation aligns with evidence that osmotic and ionic stimuli regulate AQP1 membrane trafficking and glycosylation status [14,42]. However, without membrane-fraction analyses or functional water-permeability assays, the physiological significance of these glycosylated forms, whether adaptive, injurious, or edema-promoting, remains uncertain.

The correlation analysis supported the tissue-specific aquaporin profiles identified by immunoblotting and extended our previous ANH findings [21]. In cardiac tissue, AQP1 showed inverse correlations with cleaved caspase-3, NF-κB p65, and TNF-α, and a positive correlation with Hsp70, suggesting reduced apoptotic and inflammatory signaling alongside an enhanced stress-response profile. This pattern aligns with reports of AQP1-mediated anti-apoptotic effects in smooth muscle cells [45], although opposing findings in myocardial infarction models indicate that AQP1-associated protection is context-dependent [46]. In contrast, cardiac AQP3 correlated positively with apoptotic and oxidative markers (Bax/Bcl2, cleaved caspase-3, 4-HNE) and negatively with Hsp70 and Hsp90, consistent with its established role in facilitating H_2_O_2_ transport and redox-dependent signaling [47,48]. In the aorta, the correlation pattern was largely inverse: AQP1 and AQP3 were associated with lower apoptotic, inflammatory, and oxidative-damage markers, while AQP4 showed inverse correlations with 4-HNE and AGE but a positive association with Hsp90. The positive AQP4–Hsp90 correlations may suggest coordinated stress responses, although their functional significance remains to be clarified [49]. Importantly, these correlation patterns complement the detection of glycosylated AQP1 and AQP3 isoforms, suggesting that aquaporin regulation during ANH involves coordinated changes in expression, post-translational modification, and stress-injury coupling rather than a uniform response across tissues. Importantly, these correlations were calculated using pooled NS-ANH and RL-ANH data; therefore, some of the observed associations may partly reflect between-group differences rather than relationships within individual treatment groups. In addition, as the *p* values were not adjusted for multiple correlation testing, these findings should be interpreted with appropriate caution.

This study has limitations. Histopathological examination, including H&E staining and tissue injury scoring, was not performed, limiting morphological confirmation of cell death, inflammatory cell infiltration, and structural tissue damage. Furthermore, the single 24 h time point cannot distinguish transient adaptation from persistent injury or recovery. Whole-tissue immunoblotting cannot resolve cell-specific responses or membrane localization, which is critical because AQP1 can rapidly translocate to the membrane and the total protein levels may not reflect water transport [42]. Future studies should incorporate multiple time points, cell-specific immunolocalization, membrane-fraction analyses, functional water-permeability measurements, and assessments of tissue water content, histological injury, and cardiac and vascular function. Clinically, linking these molecular profiles to edema, endothelial dysfunction, and organ injury may help determine whether NS and RL should be selected based on physiological context and organ vulnerability during ANH. Because recent clinical evidence suggests modest average differences between balanced crystalloids and saline that vary across patient populations [28,50], our results provide preliminary insights into fluid-dependent molecular responses, which warrant further investigation.

## 4. Materials and Methods

### 4.1. Ethics Statement and Animals

Twenty-four male Han:SPRD rats (6 months old, 400–500 g) were used in this study. Animals were housed under controlled environmental conditions (12 h light/dark cycle, 60% humidity, 20–24 °C) with ad libitum access to food and water. Ethical approval was provided by the Animal Ethics Committee of the University of Health Sciences, Bağcılar Training and Research Hospital, Istanbul, Türkiye (approval no: HADYEK-2024/20; 29 March 2024). All animal care and experiments were conducted at the Istanbul Bağcılar Training and Research Hospital Experimental Research and Skill Development Training Centre (BADABEM), in accordance with ARRIVE guidelines and the National Research Council’s Guide for the Care and Use of Laboratory Animals. All efforts were made to minimize animal discomfort.

### 4.2. Experimental Setup and ANH Model

Animals were randomly assigned to four experimental groups using the RAND() function in Microsoft Excel: (1) control group (*n* = 6), which received no intervention; (2) sham group (*n* = 6), which underwent anesthesia only; (3) normal saline (NS)–ANH group (*n* = 6), in which ANH was performed using 0.9% sodium chloride, and (4) Ringer lactate (RL)–ANH group (*n* = 6), in which ANH was performed using Ringer Lactate for volume replacement.

Rats in the sham and ANH groups were anesthetized with 2% sevoflurane in a gas mixture of 50% oxygen and 50% air. Before the procedure, body weight was recorded, and the estimated blood volume (EBV) was calculated. The EBV of rats was assumed to be 64 mL/kg [51], corresponding to ~5–7 mL/100 g body weight [52]. Animals were positioned supine, and 26-gauge catheters were inserted into the tail vein and the left femoral artery. The blood volume to be withdrawn during ANH was determined using the following formula: Allowable blood volume (mL) = EBV × [(HCT_current_ − HCT_target_)/HCT_average_] [53].

The volume of blood withdrawn during ANH corresponded to approximately one-third of the total blood volume in adult rats. A similar proportion is observed in humans: removing four units of whole blood (~450 mL per unit; ~1.8 L total) from an adult with an estimated blood volume of 5 L also represents roughly one-third of the circulating volume, resulting in marked hemodilution. This experimental approach therefore reflects a deep hemodilution model, a strategy infrequently used in clinical practice [54,55,56] yet consistent with hematocrit reductions reported in other studies employing profound hemodilution [29,57]. Such a model was selected to characterize early molecular responses triggered by substantial intravascular dilution.

**Figure 6:**
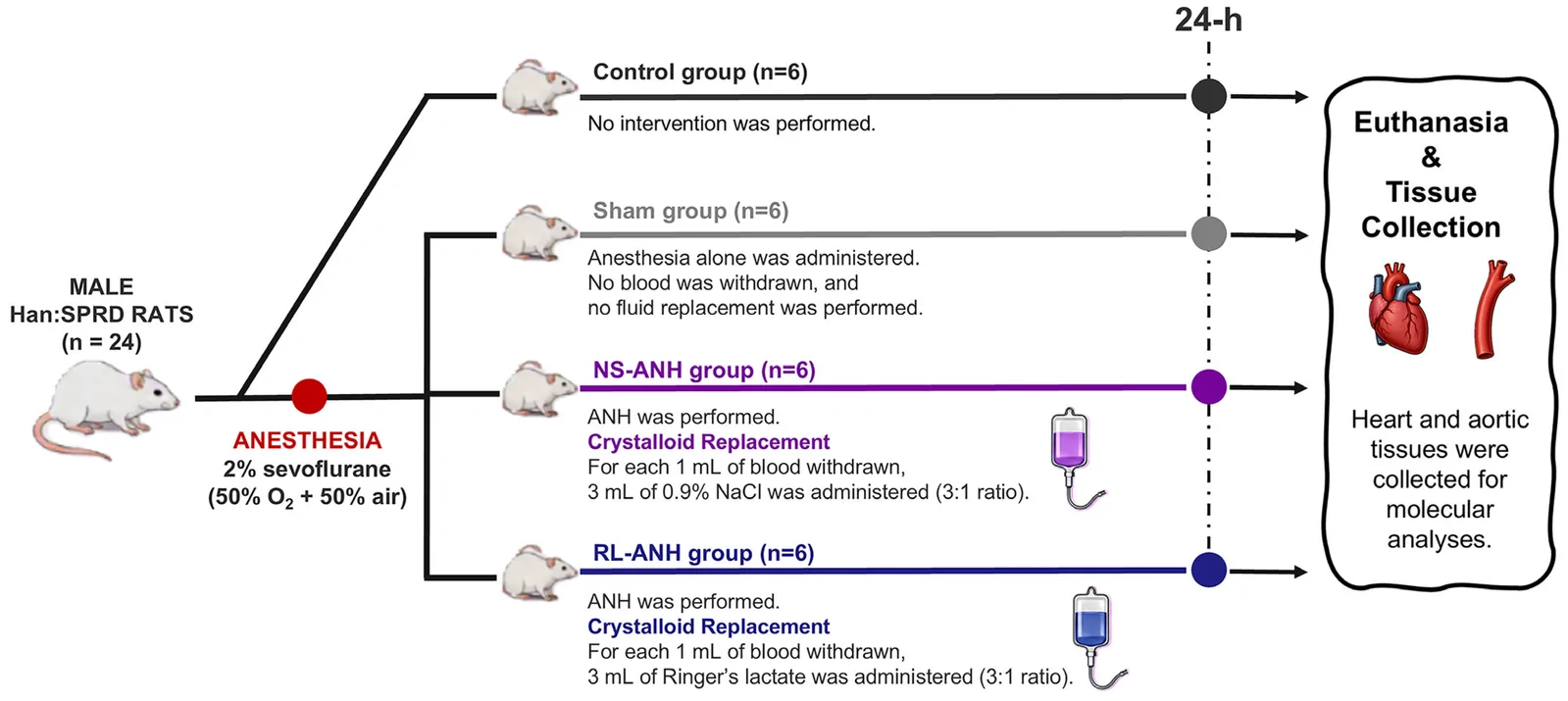
Experimental design of crystalloid-dependent acute normovolemic hemodilution (ANH). Twenty-four male Hannover–Sprague Dawley rats (*n* = 6/group) were assigned to control, sham, normal-saline (NS)-ANH, or Ringer’s-lactate (RL)-ANH. Control animals received no intervention. Sham animals underwent anesthesia with 2% sevoflurane in 50% O_2_/50% air without blood withdrawal or fluid replacement. NS-ANH and RL-ANH groups underwent ANH under identical anesthetic conditions. For each 1 mL of blood withdrawn, 3 mL of either 0.9% NaCl or Ringer’s lactate was infused (1:3 blood-to-crystalloid ratio). After 24 h, animals were euthanized, and heart and aortic tissues were collected for molecular analyses.

The target hematocrit used to calculate the blood withdrawal volume was about 25%. Blood was withdrawn via the femoral artery. To maintain normovolemia, crystalloid replacement was administered intravenously (i.v. injection) at a 3:1 ratio (3 mL of crystalloid for every 1 mL of blood removed). Ringer’s lactate (Polifarma, Istanbul, Türkiye) is a balanced crystalloid encompassing sodium (129.4 mmol/L), potassium (5.4 mmol/L), calcium (4 mmol/L), chloride (112 mmol/L), and lactate (27.8 mmol/L); in contrast, normal saline (Polifarma) is composed of 0.9% sodium chloride. The hemodilution procedure was completed in approximately 30 min, comprising 10 min of blood withdrawal followed by 20 min of crystalloid administration, which began only after blood withdrawal was completed. The withdrawn blood was not reinfused during the 24 h observation period or before tissue collection. Arterial blood samples (1 mL) were collected before and 24 h thereafter to measure hematocrit (HCT) using an ABL90 FLEX PLUS blood gas analyzer (Radiometer, Copenhagen, Denmark). At 24 h after ANH, animals were deeply anesthetized with intravenous ketamine and euthanized. Heart and aortic tissues were then harvested and stored for molecular analyses (Figure 6).

### 4.3. Protein Extraction from Tissues and Western Blot Analysis

Protein isolation and immunoblotting were performed according to established procedures [21]. Heart and aorta tissues were immediately snap-frozen in liquid nitrogen and finely pulverized with a chilled mortar and pestle. Aorta homogenates were prepared in 500 μL of TRIzol reagent (Invitrogen, Carlsbad, USA) following the manufacturer’s protocol. Heart tissues were homogenized in ice-cold lysis buffer containing 50 mM Tris-HCl (pH 7.6), 150 mM NaCl, 1% NP-40, 1% Triton X-100, 10% glycerol, 1 mM PMSF, and an EDTA-free protease inhibitor cocktail.

All homogenates underwent methanol–chloroform precipitation to obtain purified protein fractions [58]. Protein concentrations were determined by BCA assay (iNtRON Biotechnology, Seongnam, Republic of Korea). Equal amounts of protein (30 μg per lane) were separated by SDS-PAGE under reducing conditions and transferred to PVDF membranes (Bio-Rad, Hercules, USA). Membranes were blocked with 5% non-fat dry milk in TBST and incubated overnight at 4 °C with the appropriate primary antibodies (Table 2). After TBST washes, membranes were incubated with HRP-conjugated secondary antibodies for 2 h at room temperature. β-actin served as the loading control. Protein bands were visualized with enhanced chemiluminescence substrate (Thermo Scientific, Kwartsweg, Holland), captured with a ChemiDoc XRS imaging system (Bio-Rad), and quantified using ImageLab 6.0.1 software.

The following molecular targets were analyzed: apoptosis-associated proteins (Bax, Bcl2, cleaved caspase-3), inflammatory signaling markers (NF-κB p65, TNF-α), aquaporins (AQP1, AQP3, AQP4), heat-shock proteins (Hsp70, Hsp90), lipid peroxidation markers (4-HNE, MDA), and AGE.

### 4.4. Statistical Analysis

Statistical analyses were performed using GraphPad Prism v11.0.2. Normality was assessed using the Shapiro–Wilk test. Table 1 presents data as the median (interquartile range [IQR], 25th–75th percentiles), whereas the graphs show molecular data as mean ± standard deviation (SD). Hematocrit measurements were analyzed using two-way ANOVA with group and time as factors, followed by Tukey’s post hoc test. Other molecular responses were analyzed using one-way ANOVA, followed by Tukey’s post hoc test. Correlation analyses were performed using pooled NS-ANH and RL-ANH data (*n* = 12). Spearman’s rank correlation coefficients were interpreted as weak (r = 0.20–0.39), moderate (r = 0.40–0.59), strong (r = 0.60–0.79), or very strong (r = 0.80–1.00). *p* values were not adjusted for multiple correlation testing. Statistical significance was set at *p* < 0.05.

## 5. Conclusions

In conclusion, replacement-fluid composition modulated the early cardiovascular tissue response to ANH. Compared with NS-ANH, RL-ANH showed lower apoptosis-associated marker levels in both tissues, along with lower inflammatory and lipid-peroxidation markers, particularly in the aorta. However, cardiac lipid peroxidation and Hsp70 responses were similar between fluids, whereas RL-ANH produced greater Hsp90 expression and distinct tissue-specific AQP remodeling, including glycosylated AQP1/AQP3 species, indicating that the response was neither uniform nor attributable to a single pathway. Collectively, these findings suggest a comparatively more favorable molecular profile with RL-ANH, particularly in aortic tissue. Further studies incorporating cell-specific and functional AQP analyses, and direct assessments of edema, histological injury, cardiac performance, and vascular function are required to determine more clearly whether these molecular and tissue-level differences translate into clinically meaningful advantages and to clarify the potential superiority of replacement fluids in both experimental and clinical ANH settings.

## Figures and Tables

**Table 1:** Baseline characteristics and hematocrit values.

		Control	Sham	NS-ANH	RL-ANH	*p* Value
**Weight (g)**	**Median (IQR)**	447.5 (438.8–470)	455 (422.5–487.5)	452.5 (437.5–470)	467.5 (407.5–510)	0.9993
**Estimated blood volume (mL)**	**Median (IQR)**	28.82 (26.76–31.23)	27.85 (24.73–31.38)	29.57 (26.29–32.53)	29.01 (26.57–30.26)	0.2417
**Collected blood volume (mL)**	**Median (IQR)**	N/A	N/A	11.80 (10.40–12.75)	11.43 (10.37–12.45)	0.7345
**Hematocrit (HCT, %)**						
**0 h (baseline)**	**Median (IQR)**	39.50 (38–42.25)	39.50 (38.75–41)	39 (37.75–41)	40 (38–41.25)	0.8442
**24 h (after model processing)**	**Median (IQR)**	41 (38.50–42.50)	38 (37–39.75)	24.50 (23.50–26.25)	26.50 (23.75–28.25)	<0.0001 ****
	***p* Value**	0.5543	0.3030	<0.0001 ****	<0.0001 ****	

**** *p* < 0.0001.

**Table 2:** Antibodies used for western blotting.

Antibody	Host	Dilution	Catalog Number	Company *
Anti-AQP1	Rabbit	1:1000	AF5231	Affinity Biosciences
Anti-AQP3	Rabbit	1:1000	AF5222	Affinity Biosciences
Anti-AQP4	Rabbit	1:500	sc-20812	Santa Cruz Biotech.
Anti-Bax	Mouse	1:1000	NBP1-28566	Novus Bio.
Anti-Bcl2	Rabbit	1:1000	NB100-56098	Novus Bio.
Anti-Cas-3^pro&cleaved^	Rabbit	1:1000	AF6879	Affinity Biosciences
Anti-Hsp70	Mouse	1:1000	MA3-007	Thermo/Invitrogen
Anti-Hsp90	Rabbit	1:1000	MA5-33168	Thermo/Invitrogen
Anti-NF-κB p65	Rabbit	1:1000	10745-1-AP	Proteintech
Anti-TNF-α	Rabbit	1:1000	NB600-587	Novus Bio.
Anti-MDA	Mouse	1:1000	NBP2-59367	Novus Bio.
Anti-4HNE	Mouse	1:1000	Ab46545	Abcam
Anti-AGE	Rabbit	1:1000	Ab176173	Abcam
Anti-β-actin	Mouse	1:5000	MA5-15739	Thermo/Invitrogen
Anti-Mouse IgG	Goat	1:5000	31,430	Thermo/Invitrogen
Anti-Rabbit IgG	Goat	1:5000	31,460	Thermo/Invitrogen

* Abcam (Cambridge, MA, USA), Affinity Biosciences (Liyang, China), Santa Cruz Biotechnology (Dallas, USA), Novus Biologicals (St. Louis, MO, USA), Proteintech (Chicago, IL, USA), Thermo/Invitrogen (Carlsbad, CA, USA).

## Data Availability

The data that support the findings of this study are available from the corresponding authors upon reasonable request.

## Abbreviations

The following abbreviations are used in this manuscript:

4-HNE	4-Hydroxynonenal
AGE	Advanced Glycation End-products
ANH	Acute normovolemic hemodilution
AQP	Aquaporin
Bax	Bcl2-associated X protein
Bcl2	B-cell lymphoma 2
Cas3	Caspase-3
HCT	Hematocrit
Hsp	Heat shock protein
MDA	Malondialdehyde
NF-κB	Nuclear factor kappa B
NS	Normal saline
RL	Ringer’s lactate
TNF-α	Tumor necrosis factor alpha
